# Accuracy of pelvic bone segmentation for 3d printing: a study of segmentation accuracy based on anatomic landmarks to evaluate the influence of the observer

**DOI:** 10.1186/s41205-024-00237-8

**Published:** 2024-10-08

**Authors:** Lukas Juergensen, Robert Rischen, Max Toennemann, Georg Gosheger, Dominic Gehweiler, Martin Schulze

**Affiliations:** 1 Department of General Orthopedics and Tumor Orthopedics, University Hospital Muenster, 48149 Münster, Germany; 2 Clinic for Radiology, University Hospital Muenster, 48149 Muenster, Germany; 3 AO Research Institute Davos (ARI), 7270 Davos, Switzerland

**Keywords:** 3D printing, Segmentation, Accuracy, Pelvic bones, Inter-observer reliability, Landmarks, Quality assurance, Point of care

## Abstract

**Background:**

3D printing has a wide range of applications and has brought significant change to many medical fields. However, ensuring quality assurance (QA) is essential for patient safety and requires a QA program that encompasses the entire production process. This process begins with imaging and continues on with segmentation, which is the conversion of Digital Imaging and Communications in Medicine (DICOM) data into virtual 3D-models. Since segmentation is highly influenced by manual intervention the influence of the users background on segmentation accuracy should be thoroughly investigated.

**Methods:**

Seventeen computed tomography (CT) scans of the pelvis with physiological bony structures were identified, anonymized, exported as DICOM data sets, and pelvic bones were segmented by four observers with different backgrounds. Landmarks were measured on DICOM images and in the segmentations. Intraclass correlation coefficients (ICCs) were calculated to assess inter-observer agreement, and the trueness of the segmentation results was analyzed by comparing the DICOM landmark measurements with the measurements of the segmentation results. The correlation between segmentation trueness and segmentation time was analyzed.

**Results:**

The lower limits of the 95% confidence intervals of the ICCs for the seven landmarks analyzed ranged from 0.511 to 0.986. The distance between the iliac crests showed the highest agreement between observers, while the distance between the ischial tuberosities showed the lowest. The distance between the upper edge of the symphysis and the promontory showed the lowest deviation between DICOM measurements and segmentation measurements (mean deviations < 1 mm), while the intertuberous distance showed the highest deviation (mean deviations 14.5—18.2 mm).

**Conclusions:**

Investigators with diverse backgrounds in segmentation and varying experience with slice images achieved pelvic bone segmentations with landmark measurements of mostly high agreement in a setup with high realism. In contrast, high variability was observed in the segmentation of the coccyx. In general, interobserver agreement was high, but due to measurement inaccuracies, landmark-based approaches cannot conclusively show that segmentation accuracy is within a clinically tolerable range of 2 mm for the pelvis. If the segmentation is performed by a very inexperienced user, the result should be reviewed critically by the clinician in charge.

## Background

### 3d printing in medicine

3D printing has a wide range of applications and has brought a considerable transformation to many medical fields. The various solutions that 3D printing offers to improve treatment strategies can be grouped into three main categories: anatomical models, e.g. for surgical planning [1, 2], customized implants [3, 4] and patient-specific instruments [5, 6]. These capabilities can aid in medical training [7, 8], facilitate patient education [9, 10], and improve outcomes of procedures and surgeries [11, 12]. For a wide range of medical specialties, the appropriateness of 3D printing has been specifically analyzed by the Radiological Society of North America 3D printing Special Interest Group [13].

Ensuring quality assurance in the use of medical 3D printing is essential for patient safety and requires a QA program that encompasses the entire production process. This process typically starts with imaging and continues with segmentation, which is the conversion of Digital Imaging and Communications in Medicine (DICOM) data into virtual 3D-models. There are various software options available for segmentation, ranging from free software such as 3D Slicer [14] to certified medical devices such as Mimics (Materialize, Belgium). The significant manual intervention required for segmentation demands a thorough investigation of how user interaction affects segmentation results. In addition, the influence of the software used and the parameters chosen should be considered.

In the context of regulatory requirements, such as the Medical Device Regulation (MDR) for the European region, the question arises whether the advantages of freely available software can also be utilized for segmentation, and whether the use of free segmentation software in clinical research settings can enhance patient care. Possibly, without the need for high investment costs, low-threshold experience could be acquired, consequently facilitating application and technology research in regions with limited infrastructure or in healthcare facilities with limited resources [15]. However, safety and reliability of the segmentation process are essential prerequisites for this.

To assess safety and reliability, two key aspects should be considered: firstly, the influence of the software, and secondly, the qualification of the users. Given the early stage of development of medical 3D printing at the point of care, there is currently a lack of established standards for the qualification of personnel responsible for performing segmentation. In clinical practice, it is common for technicians to perform segmentation tasks. It is not clearly defined what level of anatomical knowledge and experience with multislice images is required to achieve high-quality segmentation results. The definition of high quality can vary for anatomical regions. The pelvic bones, with their variable shapes, are ideal for studying the accuracy of segmentation in a generalizable anatomical region. They include intricate structures in the sacrum and coccyx regions, as well as extensive free-form surfaces in the ilium. Furthermore, according to a recent review, pelvic bones have not yet been extensively studied in the context of quality assurance for medical 3D printing [16]. Since the time required for segmentation is a significant cost factor and often limits the feasibility of 3D printing solutions, it should also be considered.

The widely used free segmentation software 3D Slicer is used as an example to assess the influence of the user’s background on the accuracy of segmentation results. For this purpose, this study examines the inter-observer variability of landmark measurements of pelvic bone segmentations performed by different observers (precision) and compares these to the corresponding measurements derived from the underlying DICOM data sets (trueness). Landmark measurements are widely used in the literature and in this study refer to distance or angle measurements between two defined anatomical structures. They were specifically chosen with the aim of enhancing the reproducibility of the measurements. The landmarks selected are based, among other aspects, on pelvimetric measurements commonly used in obstetrics, such as the obstetric conjugate diameter [17, 18].

### The medical 3d printing process and its errors

A terminology for errors in medical 3D printing processes has been introduced in a recent study [16]. Within this terminology segmentation is the conversion of Digital Imaging and Communications in Medicine (DICOM) data into virtual 3D-models, which are usually saved as STL (Standard Tessellation Language) files. STL files represent a virtual three-dimensional surface model as a mesh that is usually approximated from small triangles of the original surface structure.

The current research shifts attention to the accuracy of the segmentation process. In this study, accuracy is described as a combined concept of trueness and precision according to the definition provided by the International Organization for Standardization (ISO) 5725–2:2019 standard [19]: “ISO 5725 uses two terms, ‘trueness’ and ‘precision’, to describe the accuracy of a measurement method. ‘Trueness’ refers to the closeness of agreement between the arithmetic mean of a large number of test results and the true or accepted reference value. ‘Precision’ refers to the closeness of agreement between test results.” According to Schulze et al. this definition is adapted to the accuracy of the segmentation process which is quantified by the combination of the segmentation error (SegE, representing trueness) and the segmentation comparison error (SegC, representing precision): They defined the SegE as the deviation between the original structure and the direct result of the segmentation process, while the SegC is defined as the precision of the segmentation process when it is performed repeatedly, e.g. by different users or with different software [16].

**Fig. 1:**
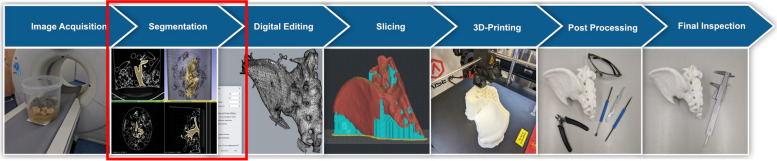
Medical 3D printing process for the production of patient specific anatomical models and its errors [16]. Highlighted with red box: focus of this study

Figure 1 shows the medical 3D printing process and the focus of this study.

## Materials and methods

### Sample size calculation

The reliability of a measurement method can be statistically measured using the Intra Class Correlation Coefficient (ICC), particularly when different observers collect quantitative data. ICC values indicate poor (≤ 0.5), moderate (< 0.5, ≤ 0.75), good (< 0.75, ≤ 0.9) and excellent (< 0.9) reliability / agreement. The inter-observer variability of pelvic bone segmentations can be quantified by ICCs, based on landmark measurements. Therefore, an ICC approach is used for the case number planning.

Bonett et al. present Eq. 1 for sample size calculation in inter-observer variability studies [20].

1 $$n=1+8*{{z}_{1-\frac{\alpha }{2}}}^{2}\frac{{\left(1-{\rho }_{plan}\right)}^{2}*{\left(1+\left(k-1\right){\rho }_{plan}\right)}^{2}}{k\left(k-1\right){{W}_{\rho }}^{2}}$$

In Eq. 1, $k$ represents the number of observers, ${W}_{\rho }$ the width of the $\left(1-\alpha \right)$ confidence interval (CI), ${\rho }_{plan}$ the planned ICC and for significance level of $\alpha =0.05$ ${z}_{1-\frac{\alpha }{2}}$ is ${z}_{\text{0,975}}=$ 1,96.

${\rho }_{plan}$ is set to 0.9 based on a systematic literature research and clinical experience as shown in the Appendix. For $k=4$ and ${W}_{\rho }=0.15$ Eq. 1 results in a case number of 17.

### Study protocol

After approval by the local ethics committee (2021–814-f-S, Ethikkommission der Ärztekammer Westfalen-Lippe und der Universität Münster, 08.02.2022) a search was performed in the database of the radiology department for CT scans of the pelvis in patients older than 18 years without evident bone pathology. Seventeen scans were randomly selected, anonymized, and exported as DICOM datasets (m:f 10:7; age, 59.1 y ± 16,6 y). These 17 cases were assigned to four observers with varying levels of anatomical knowledge, experience with multislice images, and segmentation skills. Observer 1 (O1) was an advanced medical student in his fifth year with extensive experience in segmentation using 3D Slicer. Observer 2 (O2) was an engineer with basic anatomical knowledge and limited experience with segmentation and multislice images. Observer 3 (O3) was a medical imaging expert with advanced knowledge in the field of medical image acquisition and processing. Observer 4 (O4) was a radiologist in his fourth year of residency with basic segmentation experience. O1, O2 and O4 are affiliated with the University Hospital Muenster and O3 is affiliated with the AO Research Institute, Davos. The observers are also authors of this study.

All observers were tasked with performing semi-automatic pelvic bone segmentations using 3D Slicer (version 5.0.3), including the L5 vertebra, on the 17 datasets. Additionally, the observers measured the time needed for each segmentation, from import of DICOM data sets to export of segmentation results (Ti). Further parameters were collected for the segmentations: chosen threshold value (Th), resulting export STL file size (Fi) and the number of polygons (triangles) it contains (Nu).

**Fig. 2:**
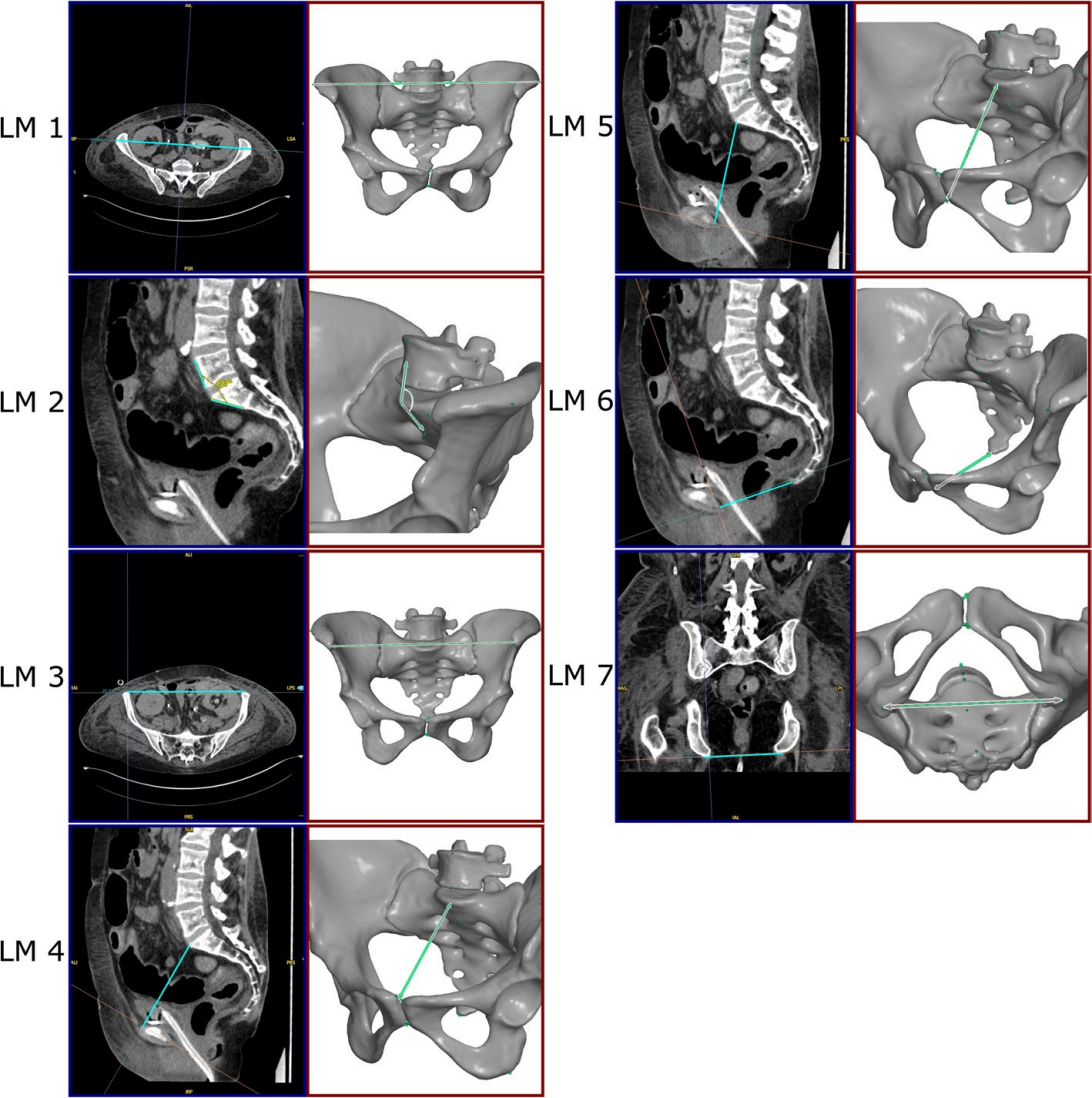
Visualization of DICOM (blue boxes, left) and STL (red boxes, right) landmark measurements. LM1: Longest distance between the iliac crests, LM2: Angle between the anterior surface of the fifth lumbar vertebra (L5) and the anterior surface of the first sacral vertebra (S1), LM3: Distance between the anterior superior iliac spines, LM4: Distance between the upper edge of the symphysis and the promontory, LM5: Distance between the lower edge of the symphysis and the promontory, LM6: Distance between the lower edge of the symphysis and the tip of the coccyx, LM7: Distance between the ischial tuberosities, further information about the orientation of the measurement planes can be found in Fig. 10 of the Appendix

A series with a slice thickness of 1.5 mm and a soft tissue kernel was pre-selected for segmentation. Preliminary experiments have shown that segmentations performed on images based on a hard reconstruction kernel are more prone to artifacts. A video tutorial for pelvic bone segmentation using 3D Slicer was provided [21], in order to assist the observers and to ensure a standardized methodology (selection of a threshold value to define a mask, followed by initial manual segmentation, *grow from seed* interpolation, removal of remaining artefacts and export). The segmentations were exported in standard tessellation language (STL) file format and seven landmarks were measured in each STL file using GOM Inspect (2022, Service Pack1, GOM, Germany). Figure 2 shows the definitions of the seven landmarks.

All landmark measurements were performed by a single observer to prevent inter-rater variability. Nevertheless, landmark identification could be challenging in some cases, so test–retest reliability was also determined. To do so, the landmarks LM1-LM7 of four cases were measured a second time by the same observer with an interval of one month and the ICCs (two-way mixed effects model for absolute agreement and single rater/measurement as described by Koo et al. [22]) were calculated individually for each landmark based on these two measurements. Assuming that conducting the measurements would lead to a training effect in landmark identification, the two cases measured first and last (pelvis_IDs 062_01, 063_01, 078_01 and 079_01) were selected for the test–retest reliability analysis.

Corresponding landmark measurements were also taken directly on the slice images using the oblique multiplanar reformation function of the Picture Archiving and Communication System (PACS) intended for diagnostics at the University Hospital Muenster (Universal Viewer, Ge Healthcare, Germany).

Analogously to the STL landmark measurements, the test–retest reliability was also determined for the landmark measurements on the DICOM data sets.

**Fig. 3:**
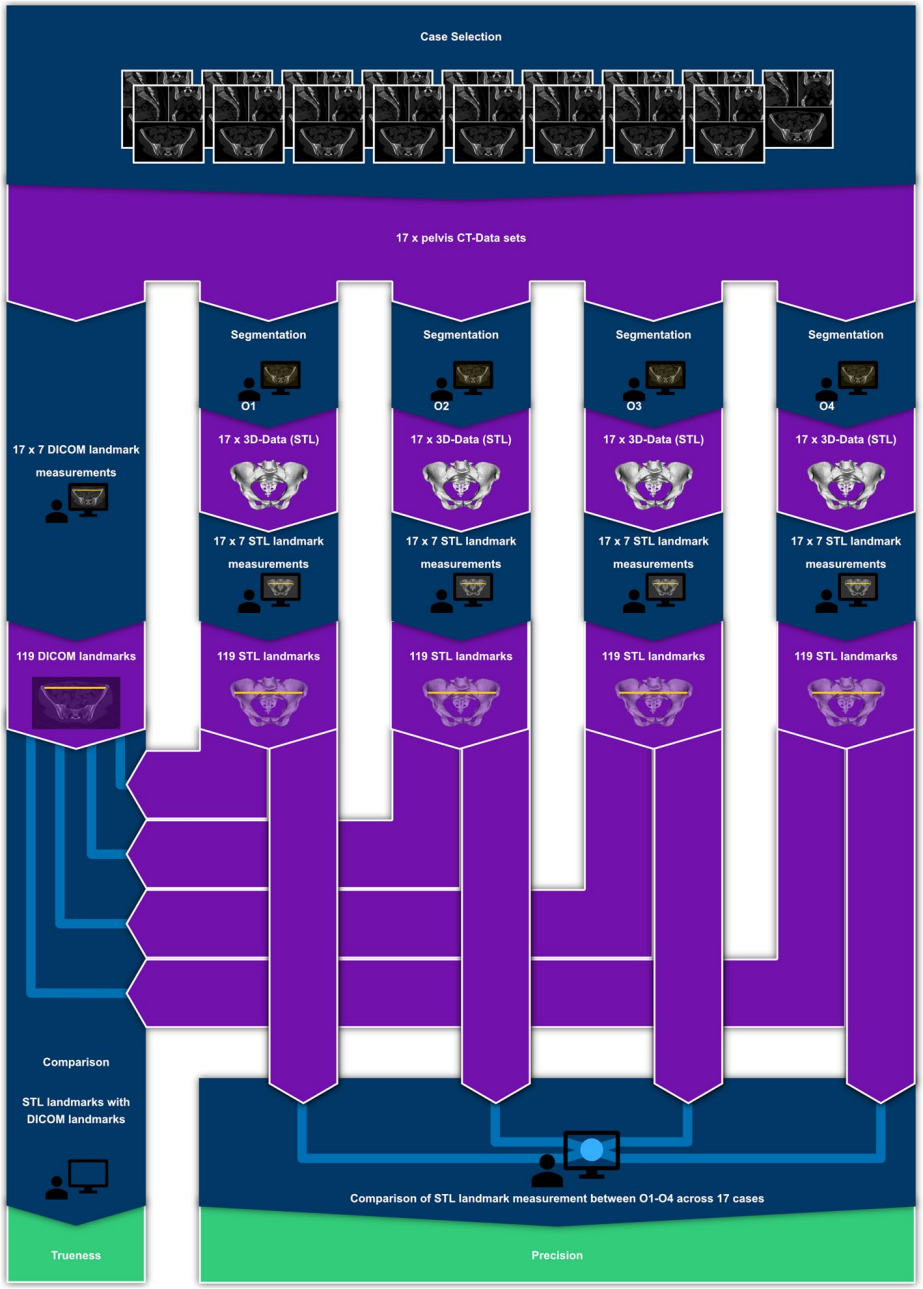
Study protocol flow chart. Segmentation: performed with 3D Slicer

For each case and observer, seven landmarks were measured in the STL files resulting in 119 measurements per observer and a total of 476 measurements across all observers. All statistical analyses were performed with SPSS Statistics (Version 29.0.0.0. (241), IBM Corp, USA). Figure 3 illustrates the study protocol.

### Statistical analysis precision

In the context of the medical 3D printing process, precision of an individual sub-step is assessed by comparing multiple results when performed repeatedly. As defined by Schulze et al. [16], the segmentation comparison error (SegC) represents the precision of the segmentation. In this study, it reflects the variability caused by the influence of observers with different backgrounds.

The mean values of each landmark were calculated across the 17 cases and four observers. In addition, mean landmark values were calculated individually for each of the four observers. Of these, the minimum and maximum values are reported. Additionally, the focus was on the four key parameters segmentation time (Ti), threshold (Th), file size (Fi) and number of polygons (Nu).

To assess the inter-observer variability of the pelvic bone segmentations, the two-way random effects model for absolute agreement and single rater/measurement of the Intra Class Correlation Coefficient (ICC), as described by Koo et al. [22], was determined for each of the landmarks as well as for the parameters Th, Ti, Fi and Nu.

Beyond the ICC, the difference between the highest and lowest measurement of each case and landmark, representing the range across the four observers (O1-O4), was calculated according to Eq. 2. In Eq. 2, ${STL}_{{LM}_{m }}max$ represents the highest of four STL measurements of landmark ${m}$ for a given case, while ${STL}_{{LM}_{m }}min$ represents the lowest value.

2 $${\Delta }_{{LM}_{m}}[range]={STL}_{{LM}_{m }}max-{STL}_{{LM}_{m }}min$$

Then, the mean, maximum and minimal values, as well as the standard deviation of the range values, were computed across all cases for each landmark.

### Statistical analysis trueness

In the context of the medical 3D printing process, the trueness of an individual sub-step is determined by the deviation between the reference and the result of a sub-step. According to the definition of Schulze et al. [16], the trueness of the segmentation process can be determined by calculating the difference between the original structure or the slice images and the segmentation results (SegE). In this study, linear landmark measurements on DICOM data sets are the reference, while corresponding linear measurements in STL files represent the results of the segmentation.

According to Eq. 3 the differences between the 119 STL file measurements of each observer and the corresponding DICOM landmark measurements were calculated, representing the segmentation error (SegE, segmentation trueness) of each observer and landmark. In Eq. 2, ${STL}_{{O}_{n}{LM}_{m}}$ is the measurement of landmark $m$ in the STL file representing the segmentation result of observer $n$. ${DICOM}_{{LM}_{m}}$ is the measuremt of landmark $m$ on the DICOM data set.

3 $${\Delta }_{{O}_{n}{LM}_{m}}[trueness]={STL}_{{O}_{n}{LM}_{m}}-{DICOM}_{{LM}_{m}}$$

The mean, the minimal and the maximal difference values were calculated for each observer and landmark.

The agreement between the DICOM landmark measurements and the STL landmark measurements was assessed by Bland Altmann analysis to evaluate the trueness of the pelvic bone segmentations, to define limits of agreement and to visualize the segmentation error.

Finally, the correlation between segmentation time and segmentation trueness was analyzed to assess whether longer segmentation times correlate with a higher segmentation accuracy.

## Results

### Test–retest reliability

To analyze the test–retest reliability of the DICOM landmark measurements, LM1-LM7 of four cases were measured a second time by the same observer with an interval of one month.

Table 1 shows the results of the test–retest reliability analysis of the DICOM landmark measurements.

**Table 1:** Test–retest reliability of DICOM measurements. DICOM landmark measurements of four cases that were performed a second time by the same observer with an interval of one month between the measurements. mean m1: mean of first measurement, mean m2: mean of second measurement, range min: lowest difference between the two measurements, range max: highest difference between the two measurements, range mean: average difference between the two measurements ICC: intra class correlation coefficient, CI: confidence interval

	mean		range				ICC^a^ 95% CI	
landmark	m1	m2	min	max	mean	ICC^a^	lower limit	upper limit
LM1 [mm]	290.2	291.0	0.6	3.4	1.7	0.995	0.947	1.000
LM2 [°]	124.7	124.2	0.8	3.7	2.6	0.984	0.789	0.999
LM3 [mm]	232.3	231.1	2.2	3.7	2.9	0.980	0.808	0.999
LM4 [mm]	112.2	114.2	0.1	3.6	2.0	0.984	0.592	0.999
LM5 [mm]	120.2	121.4	0.6	2.4	1.6	0.988	0.806	0.999
LM6 [mm]	94.9	93.6	0.1	8.3	2.8	0.887	0.092	0.992
LM7 [mm]	107.4	110.3	2.3	9.5	4.7	0.881	0.240	0.992

^a^Two-way mixed effects, absolute agreement, single rater/measurement

To analyze the test–retest reliability of the STL landmark measurements, LM1-LM7 of four cases were measured a second time by the same observer with an interval of one month. Sufficiently reliable results were observed for the landmarks LM1, LM2 and LM4-LM6. The measurements of LM7 showed the lowest reliability. Table 2 shows the results of the test–retest reliability analysis of the STL landmark measurements.

**Table 2:** Test–retest reliability of STL measurements. STL landmark measurements of four cases that were performed a second time by the same observer with an interval of one month between the measurements**.** mean m1: mean of first measurement, mean m2: mean of second measurement, range min: lowest difference between the two measurements, range max: highest difference between the two measurements, range mean: average difference between the two measurements ICC: intra class correlation coefficient, CI: confidence interval

	mean		range				ICC^a^ 95% CI	
landmark	m1	m2	min	max	mean	ICC^a^	lower limit	upper limit
LM1 [mm]	288.2	287.6	0.2	2.5	0.9	0.998	0.980	1.000
LM2 [°]	126.2	126.1	0	1.2	0.5	0.999	0.988	1.000
LM3 [mm]	230.8	232.3	1.2	6.6	3.1	0.961	0.640	0.997
LM4 [mm]	113.1	112.3	0	1.8	0.9	0.997	0.961	1.000
LM5 [mm]	121.3	120.7	0.1	1.8	0.9	0.994	0.945	1.000
LM6 [mm]	97.9	98.1	0.7	1.9	1.4	0.991	0.882	0.999
LM7 [mm]	117.4	131.0	5.4	28.4	13.6	0.648	-0.148	0.970

^a^Two-way mixed effects, absolute agreement, single rater/measurement

Note that the segmentation of the pelvis 070_01 of observer 4 is excluded from all analyses. During the evaluation it was noticed that observer 4 accidentally performed the segmentation of the pelvis 071_01 twice and incorrectly named one of the two variants 070_01.

### Precision of the pelvic bone segmentations

In general, the precision of the segmentation process can be determined by comparing multiple segmentations with each other, e.g. performed by different observers or using different software. In the present study, the precision of the segmentation provides a measure of the dimensional differences between the segmentations performed by various observers with different backgrounds and experience in medical imaging and segmentation. The overall mean value, the maximal and minimal mean value per observer as well as the ICC are shown for the landmarks LM1-LM7 in Table 3.

**Table 3:** STL landmark measurements, mean values and ICCs. mean all: mean value across all observers and cases, min/max mean: min/max mean values per observer, ICC: intra class correlation coefficient, CI: confidence interval

					ICC^a^ 95% CI	
landmark	mean all	min mean	max mean	ICC^a^	lower limit	upper limit
LM1 [mm]	282.6	281.6	283.4	0.993	0.986	0.997
LM2 [°]	128.6	127.9	130.1	0.907	0.811	0.963
LM3 [mm]	229.0	228.3	229.6	0.992	0.983	0.997
LM4 [mm]	119.3	118.6	119.6	0.989	0.977	0.996
LM5 [mm]	129.6	129.3	129.7	0.991	0.982	0.997
LM6 [mm]	93.8	92.4	97.4	0.719	0.511	0.875
LM7 [mm]	119.8	117.8	121.3	0.847	0.712	0.936

^a^Two-way random effects, absolute agreement, single rater/measurement

The parameters Th, Ti, Fi and Nu were analyzed in addition to the dimensional accuracy. The overall mean value, the maximal and minimal mean value per observer, as well as the ICC, are shown for the parameters Th, Ti, Fi, and Nu in Table 4.

**Table 4:** Parameters Th, Ti, Fi, Nu, mean values and ICCs. mean all: mean value across all observers and cases, min/max mean: min/max mean values per observer, ICC: intra class correlation coefficient, CI: confidence interval

					ICC^a^ 95% CI	
parameter	mean all	min mean	max mean	ICC^a^	lower limit	upper limit
Th [HU]	170.1	154.6	200	0.194	0.012	0.475
Ti [min]	71.4	46.5	110.4	0.108	-0.016	0.354
Fi [kB]	37,771.8	36,038.9	40,242.7	0.702	0.469	0.880
Nu	773,611	738,065	824,387	0.703	0.469	0.880

^a^Two-way random effects, absolute agreement, single rater/measurement

**Fig. 4:**
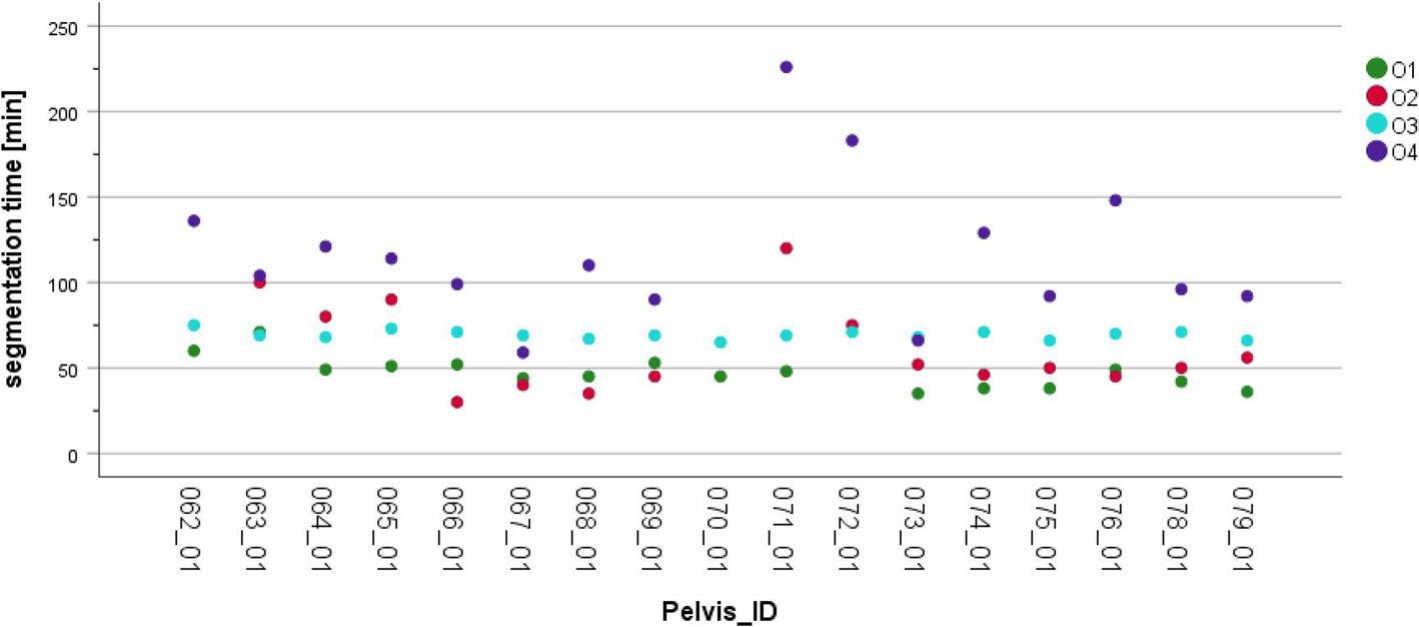
Segmentation time from import of DICOM data set to export of STL file for each case and observer. Note that four segmentation times are missing (pelvis_ID 062_01, 2*070_01 and 072_01) due to interruptions during the segmentation process

A more detailed analysis of segmentation times was conducted to assess whether the segmentations in this study contributed to a learning curve, specifically in terms of the time required to complete a segmentation. Figure 4 displays the time needed by the observers O1-O4 to complete the segmentation process. All observers processed the pelvises in the same order, starting with the lowest pelvis ID and ending with the highest.

In addition to the inter-observer reliability of the STL landmark measurements, expressed by the ICC values in Table 3, a closer look was taken at the absolute range values across the four observers.

Table 5 shows the range of the landmark measurements defined as the difference between the highest and the lowest measurement per case and landmark (Eq. 2). It includes the mean, maximal and minimal value as well as the standard deviation of the range across the 17 cases.

**Table 5:** Mean, minimal, maximal and standard deviation values of the range of the STL landmark measurements

landmark	mean range	min range	max range	standard deviation
LM1 [mm]	3.6	0.7	22.0	5.1
LM2 [°]	4.4	1.5	10.7	2.1
LM3 [mm]	2.5	0.6	11.2	2.4
LM4 [mm]	2.5	0.4	6.0	1.5
LM5 [mm]	1.9	0.6	3.9	1.1
LM6 [mm]	8.7	1.6	24.5	7.0
LM7 [mm]	12.7	4.5	25.3	6.7

### Truness of the pelvic bone segmentations

In the present study, the trueness of the segmentation provides dimensional differences between landmarks measured on DICOM data sets and in STL files.

**Fig. 5:**
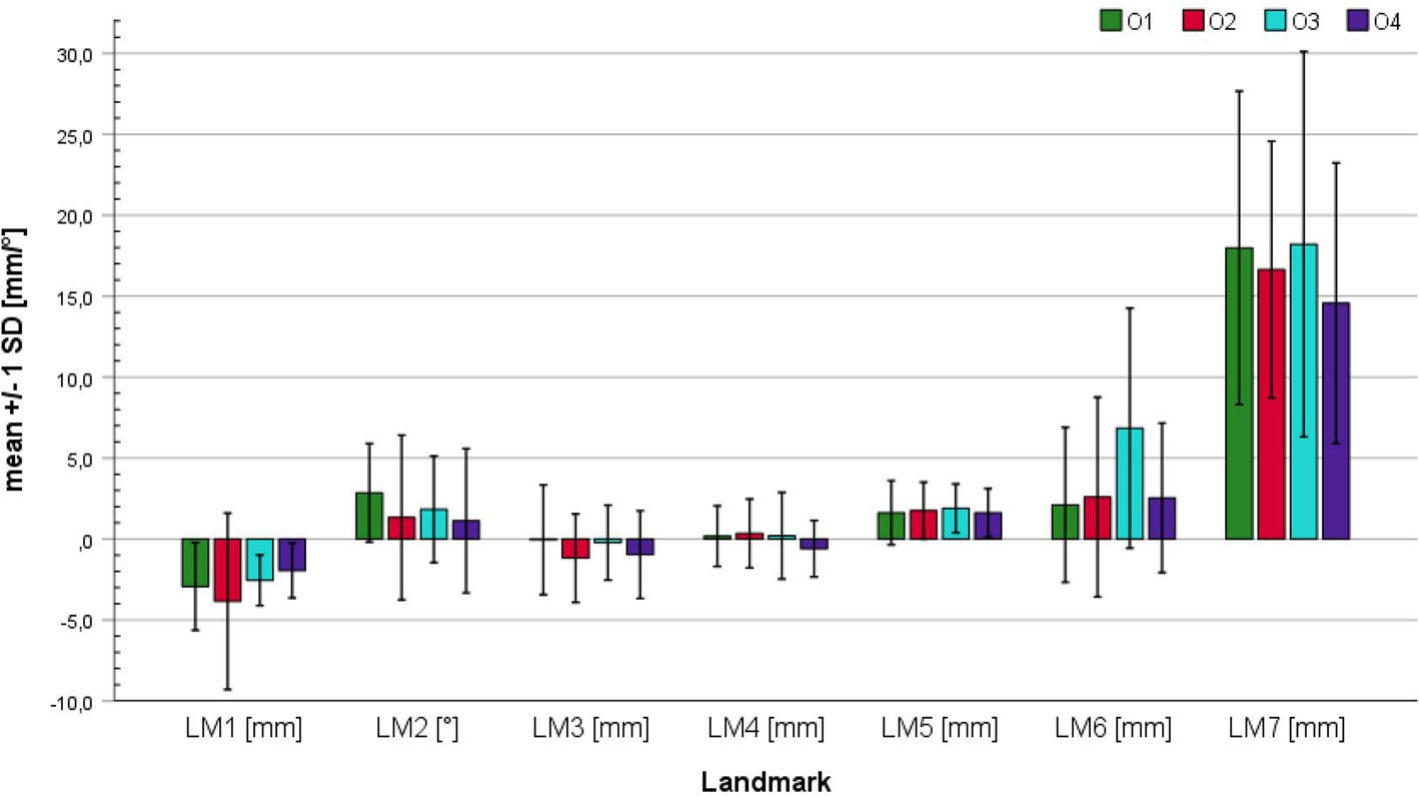
Absolute means of segmentation trueness for each landmark and observer. The error bars indicate the standard deviation. The exact values of means and standard deviations can be found in Table 7 of Appendix

**Fig. 6:**
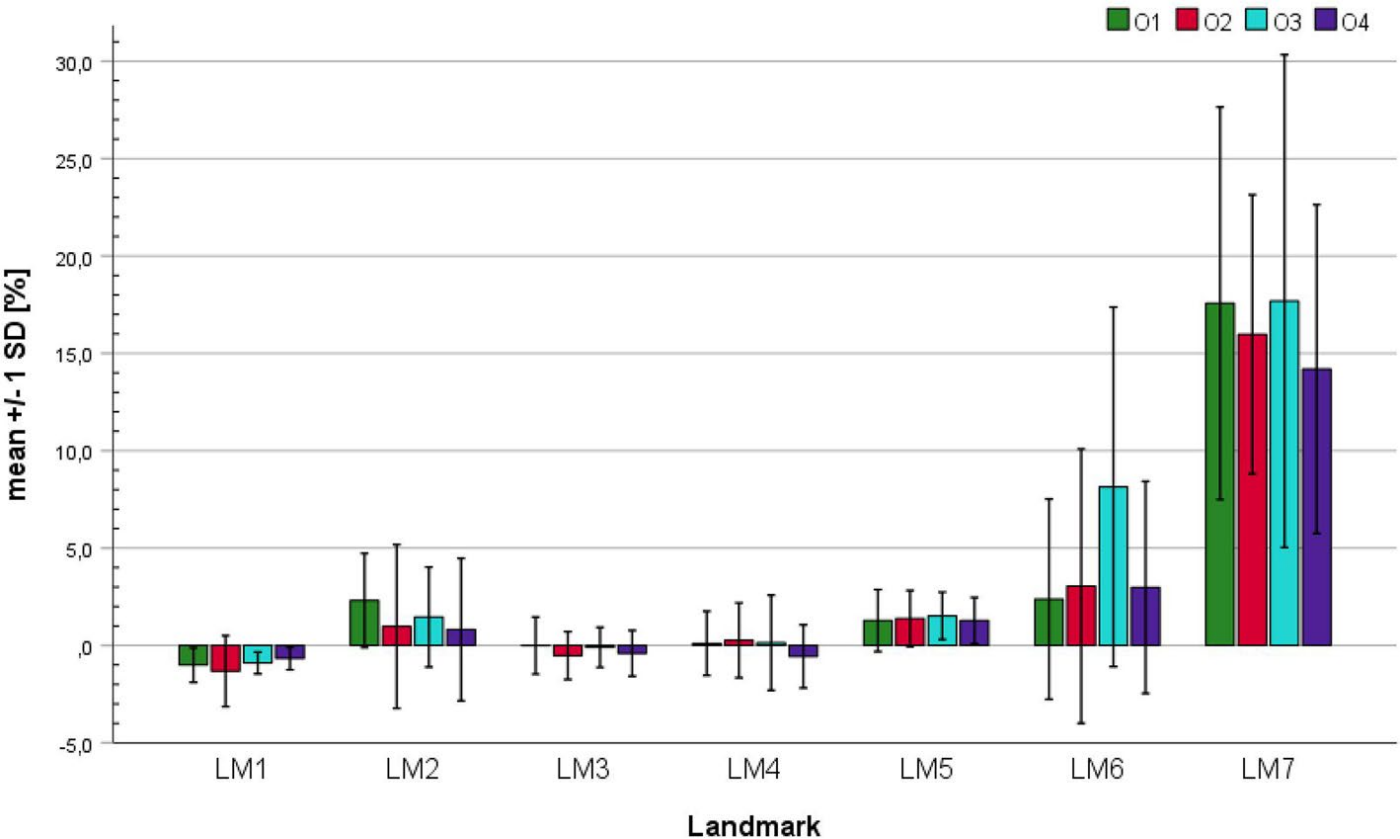
Relative means of segmentation trueness for each landmark and observer. The error bars indicate the standard deviation

Figures 5 and 6 show the mean trueness of each landmark and observer, according to Eq. 3. It should be considered that the trueness values of LM7 are of limited validity. This is due to the two effects of the wide outer contour of the ischial tuberosity: On the one hand, the test–retest reliability of the landmark measurements is not as good as for the other landmarks (Tables 1 and 2, LM 7 with poor reliability, LM1 with excellent reliability). On the other hand, it is more difficult to apply the same measurement principle to both the slice image measurements and the STL file measurements.

**Fig. 7:**
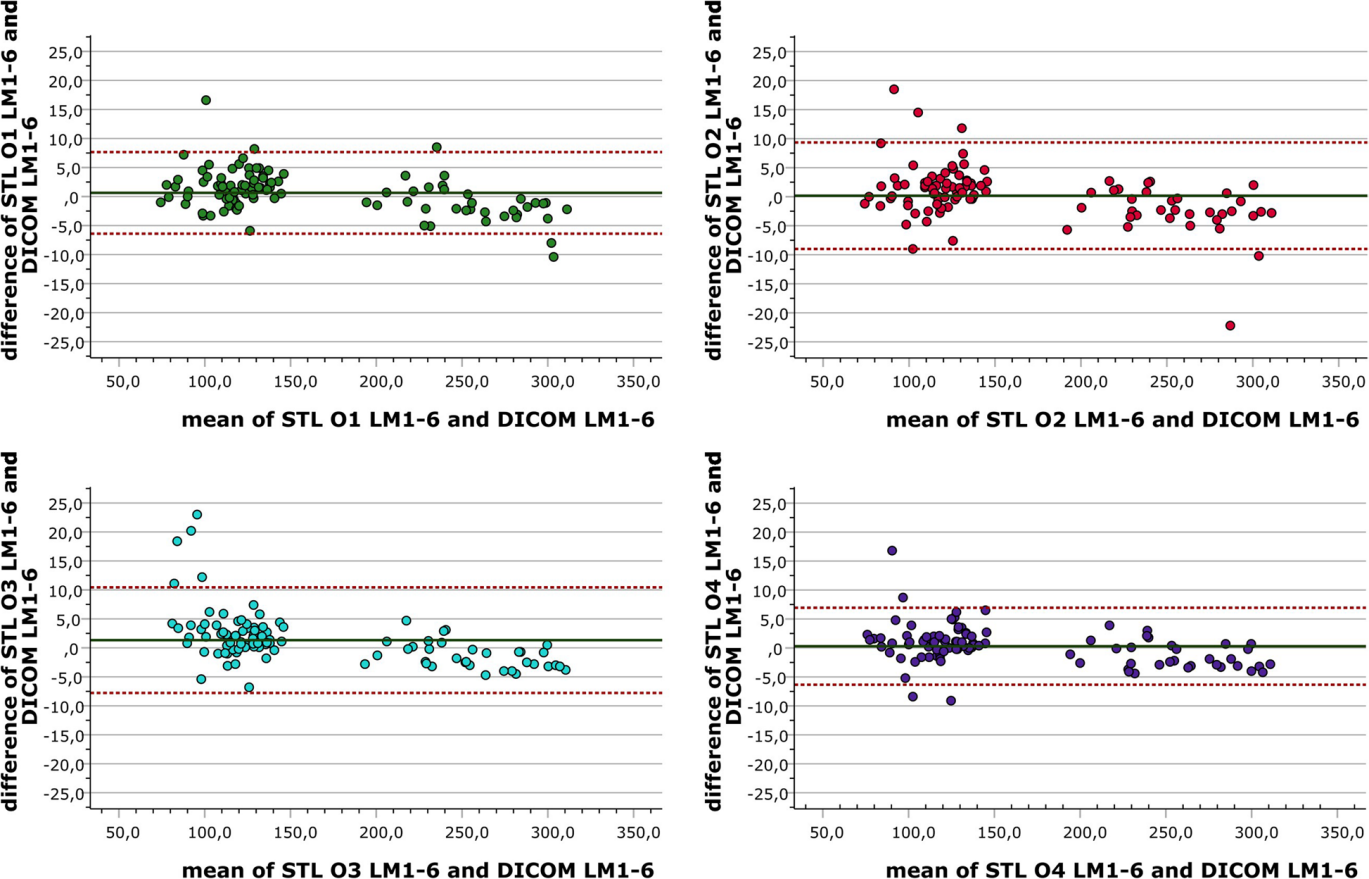
Bland Altmann plots of differences between STL and DICOM landmark measurements for each of the four observers

To visualize the trueness of the segmentation and to define the limits of agreement, Bland Altmann plots were calculated and are shown for each observer in Fig. 7. Due to the limited validity of the LM7 measurements, they are excluded from the Bland Altmann analyses and the results shown are based only on the trueness values of LM1-LM6. The mean difference, upper and lower limits of agreement (LOA) in mm are 0.64, 7.66, -6.40 for O1, 0.17, 9.34, -9.00 for O2, 1.34, 10.44, 7,78 for O3 and 0.30, 6.94, -6,34 for O4. In summary, the limits of agreement are narrowest for O1 and O4, and the fewest values outside the LOA were found for O4. For O3, almost all values outside the LOA can be attributed to the variability in the segmentation of the coccyx.

**Fig. 8:**
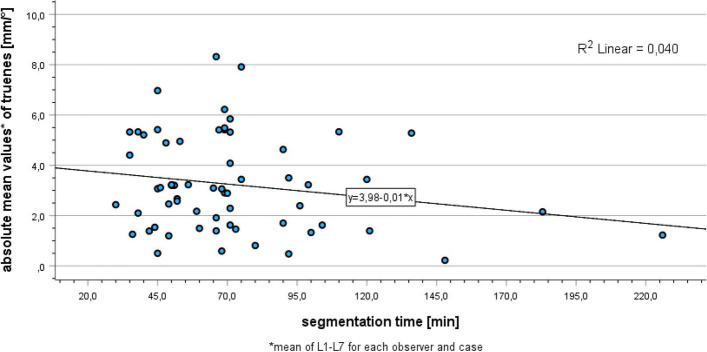
Correlation between segmentation time and absolute mean values of trueness

To assess whether longer segmentation times typically correlate with more accurate segmentation results, the correlation between the absolute mean values of trueness and the time required to complete the corresponding segmentations was analyzed. The Pearson correlation coefficient (r) is -0.199, indicating, by definition, a weak negative correlation between segmentation time and absolute mean values of trueness. However, with a p-value of 0.114, this result is not statistically significant. Figure 8 shows the correlation between the absolute mean values of trueness and the time needed to complete the corresponding segmentations together with a linear regression line. Although there might be a weak correlation between segmentation time and segmentation trueness, a poor model fit of this linear regression was found (${R}^{2}$=0.04). Consequently the segmentation trueness can not be predicted based on the segmentation time.

## Discussion

The results for the test–retest reliability of the DICOM landmark measurements showed for LM1-LM3 and LM5 good to excellent test–retest reliability based on the lower limits of the 95% CI with mean ranges of 1.6 to 2.9 mm and 2.6°, which is consistent with findings from Aubry et al. and Capelle et al. [23, 24]. In contrast, our measurements of LM4 and LM6-LM7 showed moderate to poor test–retest reliability based on the lower limits of the 95% CI with mean ranges of 2.0 to 4.7 mm. This is primarily due to the wider outer contour of the anterior superior iliac spine and the ischial tuberosity, which was described before by Keller et al. in a study on obstetric MR pelvimetry [25]. They stated that “no precise measurement point could be chosen” at the ischial tuberosity. Another aspect is relevant when looking at the test–retest reliability of the LM6 DICOM measurement: while the landmark is clearly defined, identifying the boundaries of the coccyx in cross-sectional images can be challenging. This has already been described by Keller et al. and Anderson et al. [25, 26].

The STL landmark measurements LM1, LM2 and LM4-LM6 showed good to excellent test–retest reliability based on the lower limits of the 95% CI with mean ranges of 0.9 to 1.4 mm and 1.2°, respectively. These results are consistent with findings from Jamali et al., who compared digital landmark measurements on segmented pelvises and manual measurements on pelvic models with ground truth measurements obtained with a coordinate measuring machine [27]. With deviations from the ground truth of up to 2 mm, they observed high reliability of digital measurements between different observers.

In contrast, the measurements of LM3 showed only moderate reliability, and LM7 displayed poor reliability. This could be attributed to the difficulty in precise manual landmark identification, especially in structures with a wider outer contour, such as the ischial tuberosities.

One potential solution to improve measurement accuracy in landmark-based approaches is to place artificial landmark identifiers on the target structures. Brouwers et al. used Kirschner wires to mark landmarks on the pelvis before imaging [28] and Jamali et al. employed aluminum screws for this purpose [27]. These methods significantly improve the measurement accuracy but reduce the realism of the segmentation process, as special attention is likely given to these markers during segmentation. However, aiming to evaluate the impact of the segmenters background on the segmentation results, our study opted for the highest level of realism by segmenting real patient DICOM datasets, foregoing artificial landmark identifiers.

A further alternative to improve the reliability of STL landmark measurements could be the implementation of automatic measuring algorithms. Chen et al. developed an automatic measurement system for the distal femur, which outperformed manual measurements in terms of inter- and intra-rater reliability [29].

Yet, to the best of our knowledge, similar solutions for pelvic landmark measurements in STL files are not yet available, which suggest an opportunity for future research.

Table 3 presents the mean values of the landmark measurements and their corresponding Intraclass Correlation Coefficient (ICC) values, which reflect the consistency between the segmentations of the different observers. Based on the lower limits of the 95% confidence intervals (CI), landmarks LM1 and LM3-LM4 showed excellent agreement, LM2 showed good agreement, while LM6 and LM7 showed moderate agreement. The test–retest reliability of LM6 (good) and LM7 (poor) suggests that the lower agreement for LM6 measurements is likely due to the variability of the segmentations, whereas for LM7 it is primarily due to the low reliability of the measurements themselves.

Complementing the results of this study, further research has demonstrated a high degree of agreement between segmentations conducted using various software. For example, Lo Guidice et al. presented that the percentage of the surface area within a deviation of 0.5 mm in upper airway segmentation is about 82% for 3D Slicer, compared to a range of 78% to 90% for four other segmentation software [30].

In contrast to our approach for measuring LM6, which includes the whole coccyx as a landmark, Keller et. al. and Anderson et al. measured the anteroposterior pelvic outlet, defined as the distance between the lower edge of the symphysis and the sacrum-coccyx junction [25, 26]. Nevertheless, our findings are comparable to theirs, as they also report high variability in identifying the boundaries of the coccyx. Anderson et al. report the inter-observer error of the AP outlet measurements as a standard error of 5.8 mm. Although this measure is not directly comparable to the mean range, we report for LM6 (8.7 mm), both results are of similar magnitude. Keller et al. report reliability values of 0.66 and 0.64 for the AP outlet measurements and the intertuberous distance measurements, respectively. Consistent with our findings, they found the intertuberous distance (equivalent to our LM7) to be the least reliable, and the anteroposterior outlet measurement (similar to our LM6) to be the second least reliable. They identified two different reasons for this: Due to the curved profile of the ischial tuberosity, the landmark can not be clearly defined and the measurements are greatly influenced by the examiners interpretation. In contrast, the sagittal outlet measurement is clearly defined, yet identifying the boundaries of the coccyx can be challenging. With regard to CT images, this is mainly due to the relatively low contrast of large parts of the coccyx. If pelvimetry based on automated segmentation becomes established in clinical practice, it could facilitate clinical measurements, such as those used in obstetrics. However, in clinical application, it is important to critically note that LM7 exhibited higher variability.

The level of agreement among the four observers (O1-O4), as measured through the parameters Th, Ti, Fi, and Nu is shown in Table 4. The observers were free to choose the threshold of the mask based on their individual judgment. Notably, observer O3 adopted an unique approach by consistently selecting a threshold of 200 Hounsfield units (HU) for all cases, which differed from the methods of the other observers. The difference between the highest and lowest mean threshold is 45.4 HU. However, this variation is not expected to have a significant effect on the segmentation error. This is supported by the study of Stock et al. who investigated the effect of processing techniques and threshold values on the segmentation of skeletal elements using a cadaveric immature os coxa model [31]. The maximum difference of observer selected threshold values in their study is 131 HU, resulting in a mean surface deviation of only 0.32 mm. The study by Eijnatten et al. on the impact of manual threshold selection in medical additive manufacturing further supports this [32]. The study involves a comparison between 3D-scans of dry skulls and threshold segmentations applied to CT scans of the skulls prior to dissection. Within the “multi detector CT” category, they report values of 140–185 HU for female skulls and values of 241–303 HU for male skulls. These differences of threshold values lead to a surface deviation between highest and lowest threshold models for both groups of around 0.15 mm. However, when it comes to soft tissue segmentation on CT data, the influence of thresholds must be viewed more critically, as the HU values of the target structure and adjacent tissue have a higher proximity in soft tissue segmentation.

Figure 4 illustrates the segmentation times recorded for each of the four observers. Observer O4 had the longest average segmentation time at 110.4 min, while O1 had the shortest segmentation time at 46.5 min and O3 demonstrated the least variation in segmentation times. As the observers achieved similar levels of trueness, while their segmentation times varied widely, experience with the specific segmentation software appears to be the primary factor for increasing segmentation speed, while maintaining a consistent level of quality. However, the general experience with medical imaging seems to have the greatest influence on the consistency of the segmentation workflow, which is reflected by the low variance of the segmentation times of O3. Additionally, Fig. 4 does not indicate a noticeable learning curve in segmentation time over the seventeen cases examined. On the one hand, one could conclude that the provided tutorial successfully supports the segmenters. On the other hand, a learning effect could possibly be shown with a larger number of cases and a longer observation period.

With respect to the landmark measurements, we observed good to excellent agreement for LM1-LM5 measurements. However, the parameters Fi and Nu showed poor interobserver agreement. This discrepancy suggests that the geometric accuracy of different segmentations cannot be inferred from file size and number of polygons. Due to their linear relationship, Fi and Nu showed identical ICC values (visualized in Appendix, Fig. 11).

It should also be noted that despite the good to excellent inter-observer agreement for landmark measurements LM1-LM5, clinically relevant deviation ranges are present. If clinical trueness values of up to 2 mm are acceptable in the pelvic region [33], it can be assumed that range values of up to 4 mm are clinically acceptable. However, only for LM4 and LM5, the majority of values falls within this range (based on mean + standard deviation of Table 5 < 4 mm). Relying on ICC values can be problematic when trying to infer clinically sufficient reliability from them alone. This has also been evident in other studies: Dionisio et al. compared manual and semiautomatic segmentations of bone sarcomas with each other and reported high similarity (based on dice similarity coefficients) [34]. Nevertheless, significant deviations in terms of maximal values of Hausdorff distances (a metric which is very sensitive to local deviation maxima) are reported. In this context, it is equally important to consider results such as those of Matsiushevich et al., who observed very low average deviation values when comparing different segmentation software, thus classifying the segmentation results as high quality [35]. However, they also found very significant maximum deviations. Their origin and clinical relevancy should be further evaluated.

**Fig. 9:**
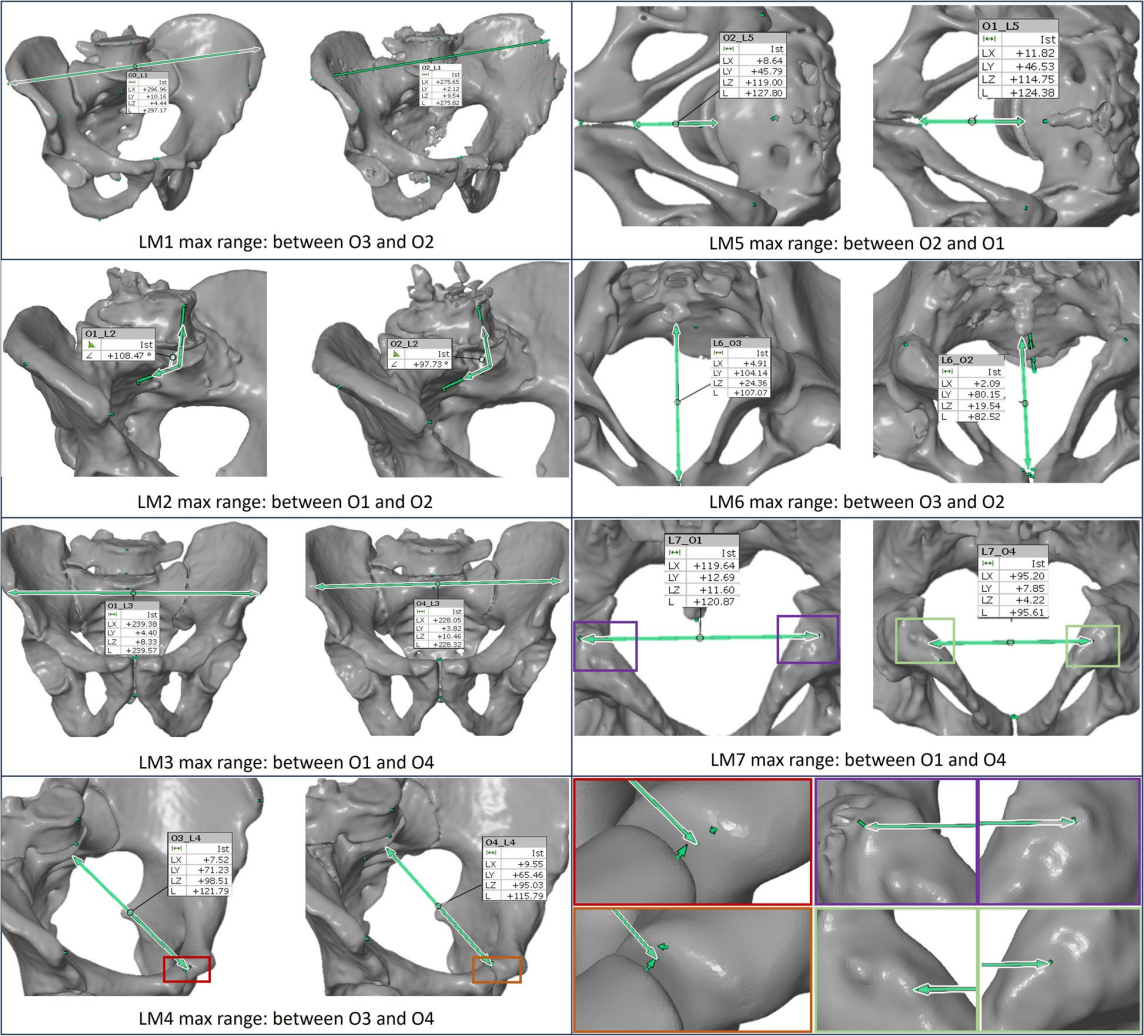
Pairs of maximal difference for each landmark LM1-LM7. Apparent differences between the segmentations cause the maximal deviations for the landmarks LM1, LM2 and LM6, whereas the maximal deviations of the landmarks LM3- LM5 and LM7 are caused primarily due to measurement inaccuracies. Bottom right: detail view of LM4 and LM7

**Fig. 11:**
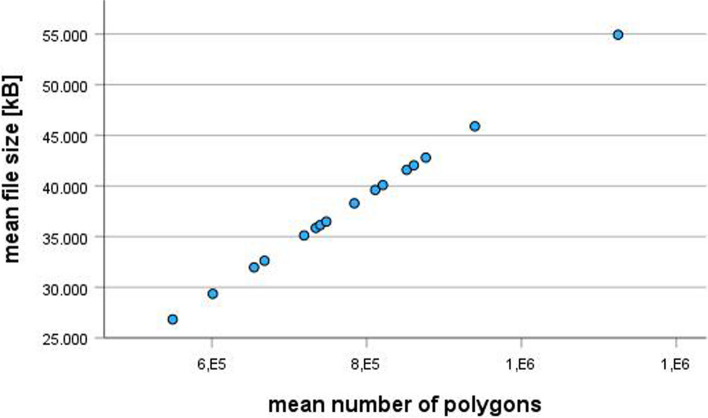
Linear correlation between export file size and number of polygons it contains

To illustrate how differences in measurements arise between the four observers, Fig. 9 visualizes the two segmentations that showed the greatest discrepancy for each landmark.

Figure 9 illustrates that the maximal deviations in landmark measurements between the four observers can be primarily attributed to three types of error: (1) inaccuracies in landmark measurements (LM3-LM5, LM7); (2) obvious segmentation errors that are easily detectable and correctable by visual inspection (LM1, LM2) and (3) inherent segmentation errors that result from the challenge of discerning boundary structures on the slice images (LM6). Regarding the first type of error, it should be taken into account that the most realistic segmentation method to assess the segmentation error necessitates the application of linear landmarks measurements, a relatively inaccurate tool [16]. The patients bones are not accessible for alternative measurement methods e.g. optical 3D-scanning. Errors that are attributed to the second type can easily be excluded in clinical practice by having a final check of the segmentation by the clinical user, adapted to the risk of intended use. Possibly also with the help of an overlay of the segmentation and the slice images. Depending on the target structures, a critical analysis should be carried out to determine which areas of the anatomy are particularly prone to errors of the third kind. These areas should be examined in more detail, and, if necessary, internal standards should be established for them.

Figure 5 shows the average trueness values for each observer and landmark, along with their standard deviations. Particularly low average deviation values were observed for landmarks LM3 to LM5, with values ranging from -1.2 to 1.9 mm. Slightly higher values were observed for LM1 and LM2, though it should be considered that, on average, LM1 is more than twice as long as LM3-LM5; consequently, the absolute error is higher at a constant relative error. As mentioned above, the deviations of LM6 are mainly due to segmentation variances, whereas LM7 is significantly influenced by measurement variability and thus has limited interpretive value. Notably, observer O3 had significantly higher deviation values for LM6 compared to the other three examiners, suggesting a different working principle used by O3 in the segmentation of the coccyx.

Salazar et al. report deviations between slice image measurements and pelvic bone segmentations performed with 3D Slicer of around 1 mm, which corresponds to the trueness they achieved with Mimics and which is consistent with our results for LM3-LM5 [36]. However, unlike our study, they only measured one landmark (from the posterior inferior iliac spine to the ischial spine), which appears to be easily identifiable.

The Bland–Altman plots in Fig. 7 provide further insight into the agreement between the segmentations and the DICOM landmark measurements for all landmarks except LM7. All observers achieved mean deviation values close to 0. Regarding limits of agreement (LOA), the best results were achieved by O4 (-6.34 – 9.94), while O3 achieved the least agreement (-7.78 – 10.44). Although most of the measurements fell within the LOA it is to be noted that the LOA significantly deviate from clinically acceptable ranges. Lo Giudice et al. found LOA from -9.86 to 9.13 cm^3^ for upper airway segmentation in their inter-observer study [30]. Unlike us, they used a volume-based approach. Their absolute values are not directly comparable to the present results, but it is noticeable that they achieved more accurate results with 3D Slicer than with Mimics.

Figure 8 and the Pearson correlation coefficient of -1.99 suggest a weak correlation between segmentation time and accuracy of segmentation results. This indicates that clinically adequate segmentation quality is attainable even with significantly reduced segmentation time, depending on the experience of the user. However, in clinical practice, there is always a compromise between segmentation time and accuracy, especially for complex structures. This has already been pointed out by Fasel et al. who demonstrated a manual segmentation time of 10 h of the sella turcica in an attempt to achieve the highest possible segmentation accuracy [37].

The focus of this publication was on segmentation accuracy, as one of the main steps of the medical 3D printing process. Yet it is crucial to acknowledge that not only the segmentation itself has an impact on the accuracy of the final printed product, but also digital editing and the physical printing. For example, smoothing, printer resolution, material properties, print bed positioning and scaling can introduce variations that affect the final printed model. These factors can, in turn, influence the haptic feedback provided by the printed model, which is one of the main advantages of 3D printing in medicine.

## Limitations

In this study, the influence of CT and segmentation parameters (such as slice thickness, kernel or threshold) was not investigated. Furthermore, landmark-based approaches are generally limited by the number of data points. This results in a relatively high proportion of structures not included in the analysis. Additionally, the measurement inaccuracy is always within the range of several millimeters. When comparing segmentation results with linear measurements on DICOM data as a reference, it should be noted that these are also associated with an inaccuracy. Optical 3D-scans can provide ground truth measurements with high accuracy and future research may use surface deviation-based methods to increase the number of measurement points. However, it should be considered that surface deviation-based approaches may reduce the degree of realism, since segmentation cannot be performed on real patient images. To significantly increase the accuracy and to complement this landmark-based approach with precise absolute error values, it can be legitimate to reduce the realism. For this purpose, a cadaver study or a substitute model could be used. In addition to this study, 3D Slicer should be tested against an approved medical device.

## Conclusion

Investigators with diverse backgrounds in segmentation and varying experience with slice images achieved pelvic bone segmentations with landmark measurements of mostly high agreement in a setup with high realism. In contrast, high variability was observed in the segmentation of the coccyx, and obvious segmentation errors (LM1 and LM2) were found in the segmentations of two cases by the inexperienced user. Deviations between the landmark measurements in the segmentations and the measurements on the slice images can be partially attributed to measurement inaccuracies. Therefore, despite the high inter-observer agreement between the four observers, landmark based approaches cannot conclusively show that segmentation trueness is within a clinically tolerable range of 2 mm for the pelvis.

If the segmentation is performed by a very inexperienced user, the result should be critically reviewed by the clinician in charge.

The experience with the specific segmentation software appears to be the primary factor for increasing segmentation speed, while maintaining a consistent level of quality. However, the general experience with medical imaging seems to have the greatest influence on the consistency of the segmentation workflow.

## Figures and Tables

**Fig. 10:**
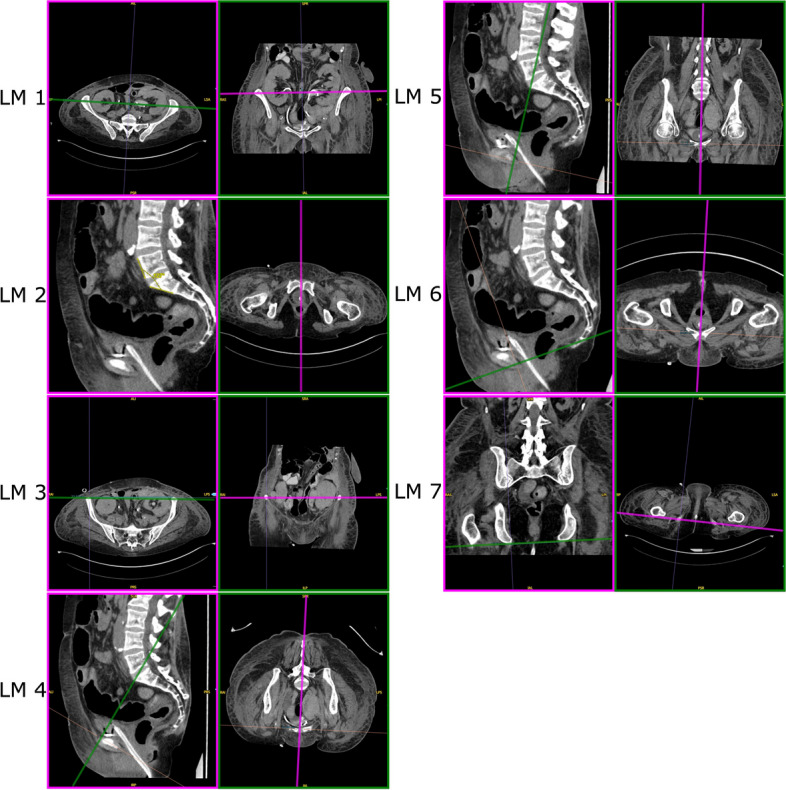
Orientation of oblique multiplanar reformations for DICOM landmark measurements

## Data Availability

The data presented in this study are available upon request from the corresponding author. The data are not publicly available due to privacy restrictions.

## Appendix

### Case number planning

${\rho }_{plan}$ Is determined on basis of clinical experience and a systematic literature research in PubMed using the following search algorithm:

(("observer variation"[MeSH Terms] OR ("observer"[All Fields] AND "variation"[All Fields]) OR

"observer variation"[All Fields] OR ("inter"[All Fields] AND "observer"[All Fields] AND "variability"[All

Fields]) AND ("bone"[All Fields] OR "bones"[All Fields]) AND ("segment"[All Fields] OR

"segmentation"[All Fields] OR "segmentations"[All Fields] OR "segmented"[All Fields] OR

"segments"[All Fields])) AND (2018:2022[pdat])

Publications since 2018 were searched for to ensure sufficient currency of the imaging technologies and the processing software. The search on January 22th, 2022 resulted in 41 search results. Five publications were found in which segmentations of bony structures are examined and evaluated using the ICC. A summary of their main findings is shown in Tables 6 and 7. Only manual and semi-automatic segmentation algorithms are included. Fully automated tools are explicitly excluded.

**Table 6:** Publications to determine ${\rho }_{plan}$

		ICC 95% CI	
publication	ICC	lower limit	upper limit
Park et al. [38]	0.91–0.98	0,79–0.97	0.96–0.99
Colombo et al. [39]	0,79–0.96	0.60–0.91	0.91–0.98
Gitto et al. [40]	74.71%-94.97% > 0,75	-	-
Imani et al. [41]	0.93–1.00	0.64–1.00	0.97–1.00
Misselyin et al. [42]	0.692–0.890	0.573–0.834	0.792–0.892

**Table 7:** Detail of segmentation trueness analysis for each observer and landmark

	N	min	max	mean	standard deviation
STL O1_LM1—DICOM LM1	17	-10.4	0.4	-2.931	2.7043
STL O1_LM2—DICOM LM2	17	-5.9	8.2	2.852	3.0382
STL O1_LM3—DICOM LM3	17	-5.1	8.5	-0.051	3.3919
STL O1_LM4—DICOM LM4	17	-3.3	3.2	0.176	1.8713
STL O1_LM5—DICOM LM5	17	-1.6	6.6	1.624	1.9791
STL O1_LM6—DICOM LM6	17	-3.3	16.6	2.110	4.7815
STL O1_LM7—DICOM LM7	17	1.4	34.2	17.984	9.6788
STL O2_LM1—DICOM LM1	17	-22.2	2.0	-3.847	5.4488
STL O2_LM2—DICOM LM2	17	-9.0	11.8	1.328	5.0851
STL O2_LM3—DICOM LM3	17	-5.7	2.7	-1.180	2.7299
STL O2_LM4—DICOM LM4	17	-4.8	3.1	0.343	2.1254
STL O2_LM5—DICOM LM5	17	-1.3	5.3	1.748	1.7604
STL O2_LM6—DICOM LM6	17	-4.4	18.5	2.592	6.1641
STL O2_LM7—DICOM LM7	17	-3.9	29.8	16.642	7.9313
STL O3_LM1—DICOM LM1	17	-4.7	0.5	-2.552	1.5656
STL O3_LM2—DICOM LM2	16	-6.8	7.4	1.829	3.2892
STL O3_LM3—DICOM LM3	17	-3.2	4.7	-0.225	2.3115
STL O3_LM4—DICOM LM4	17	-5.5	5.9	0.202	2.6713
STL O3_LM5—DICOM LM5	17	0.4	4.8	1.896	1.4999
STL O3_LM6—DICOM LM6	17	-1.0	23.0	6.849	7.4130
STL O3_LM7—DICOM LM7	17	-6.5	41.0	18.209	11.9001
STL O4_LM1—DICOM LM1	16	-4.2	0.7	-1.950	1.6885
STL O4_LM2—DICOM LM2	16	-9.1	6.5	1.125	4.4538
STL O4_LM3—DICOM LM3	16	-4.4	3.9	-0.968	2.7058
STL O4_LM4—DICOM LM4	16	-5.2	1.4	-0.599	1.7389
STL O4_LM5—DICOM LM5	16	-0.6	5.0	1.621	1.4864
STL O4_LM6—DICOM LM6	16	-2.4	16.8	2.544	4.6162
STL O4_LM7—DICOM LM7	16	-7.0	31.0	14.562	8.6645

Note that the segmentation of the pelvis 070_01 of observer 4 is excluded from the analysis, because during the evaluation it was noticed that observer 4 performed the segmentation of the pelvis 071_01 twice by mistake and incorrectly named one of the two variants as 070_01

${\rho }_{plan}$ is set to 0.9 based on a the literature research and clinical experience. For $k=4$ and ${W}_{\rho }=0.15$ Eq. 1 results in a case number of 17
